# High impact low probability events: research landscape and future opportunities

**DOI:** 10.1007/s11069-026-08107-8

**Published:** 2026-04-16

**Authors:** Lauren McMillan, Gianluca Pescaroli, Mhari Gordon, Margherita Maraschini, Silvia Torresan, José Palma-Oliveira, Beatriz Rosa, Ana Sarroeira, Benjamin D. Trump, Igor Linkov

**Affiliations:** 1 Department for Risk and Disaster Reduction, University College London, London, UK; 2 Fondazione CMCC - Centro Euro-Mediterraneo sui Cambiamenti Climatici, 73100 Lecce, Italy; 3 Factor Social, Lisbon, Portugal; 4 Faculdade de Psicologia, CIPSI, Universidade de Lisboa, Lisbon, Portugal; 5 School of Engineering, Physics and Mathematics, Northumbria University, Newcastle upon Tyne, UK; 6 University of Michigan, Ann Arbor, MI USA; 7 Carnegie Mellon University, Pittsburgh, PA USA; 8 Cambridge Centre for Risk Studies, Cambridge University, Cambridge, UK

**Keywords:** High-impact low-probability (HILP) events, Systemic risk, Resilience assessment, Risk governance, Stress testing, Complex crises

## Abstract

High-impact, low-probability (HILP) events, characterised by their extreme consequences and inherent unpredictability, pose a growing challenge to disaster risk reduction in an era of systemic and cascading crises. Traditional risk assessment frameworks, which rely on probability-based models, often fail to capture the full scope of these events, leaving critical vulnerabilities unaddressed. This semi-systematic state-of-the-art review synthesizes current academic discourse on HILPs to identify their defining characteristics and implications for disaster preparedness and response. We analyse a dataset of 109 papers, highlighting the limitations of conventional planning tools and emphasising the need for adaptive, forward-looking strategies that integrate scenario planning, stress testing, and resilience assessment. The review finds that while HILPs are increasingly recognised in national risk registers and post-disaster response frameworks, their integration into preparedness, training, and governance systems remains limited. A key gap lies in translating theoretical insights into operational strategies that can be deployed before crises occur. The paper advocates for a hazard-agnostic, interdisciplinary approach that bridges risk and resilience thinking, enabling systems to anticipate, absorb, and adapt to a broad spectrum of threats. By advancing a research agenda beyond the Sendai Framework, this review contributes to a more robust understanding of HILPs and supports the development of more resilient and responsive disaster management systems.

## Introduction

We are entering a new phase of history where complex crises are becoming business as usual. The combined effect of cascading, compound, and concurrent events is evolving systemic risk, necessitating a better understanding of critical vulnerabilities that could escalate the crisis (Pescaroli and Alexander 2018). New tools, such as stress testing, are emerging to address single points of failure across multiple threats and enhance operational capacity (Linkov et al. 2022). However, there is growing awareness that a further shift in perspective may be needed. The banking sector has suggested considering scenarios that assume “disruptions will happen” (Operational resilience: Impact tolerances for important business services, 2021). This approach is beginning to be adopted by other critical infrastructure sectors, but key elements linking theory and practice remain missing.

Central to this challenge are High-Impact, Low-Probability (HILP) events. Characterised by their extreme consequences and inherent unpredictability, HILPs do not conform to conventional risk assessment methods, which typically rely on probability-based models to estimate threats (Pescaroli et al. 2025). For example, HILPs can refer to natural hazards with low recurrence that occurred far in the past, such as major volcanic eruptions and meteorite collisions, or to recombinations of multiple hazards, such as earthquakes triggering tsunamis during particular weather conditions.

While such events have already been integrated into Risk Registers, such as the 2025 edition of the UK Government’s National Risk Register (National Risk Register—2025 edition 2025), traditional likelihood-impact matrices continue to emphasise high-probability risks. However, a traditional risk-based approach to planning for high-impact events can allow highly unlikely or unknown risks to be overlooked, resulting in even more severe outcomes (Kyrillou 2020). A lack of understanding regarding the severity and uncertainties associated with HILPs has significant implications for disaster preparedness and response, particularly as societies and infrastructure systems grow increasingly interconnected and are exposed to emergent and systemic risks (Liu and Renn 2025).

Addressing the challenges for operational capacity while maintaining resource efficiency in terms of societal resilience as a whole is often more critical than managing single threats in isolation (Pescaroli et al. 2021). Scenario building and tabletop exercises need to better integrate HILP events, which have traditionally fallen below risk appetites and thresholds for action. One critical barrier to progress is a lack of clarity regarding the subject matter itself and its implications for crisis management and disaster preparedness.

This state-of-the-art review aims to bridge this gap by exploring the academic discourse on HILP events. It seeks to answer two key questions: (1) What are the unique characteristics of HILP events that are pertinent to disaster preparedness and response? and (2) What considerations are necessary to improve understanding and preparedness for these events? By discussing the operational context of complex crises and systemic risk, with the aim to derive a research agenda going beyond the United Nations Sendai Framework for Actions (Sendai Framework for Disaster Risk Reduction 2015–2030, 2015), this review highlights the need for more adaptive, forward-looking strategies. In doing so, it lays the groundwork for a research agenda that extends beyond existing frameworks, advocating for a more robust and integrated approach to managing HILPs in an era of growing uncertainty.

## Method

### Systematic screening

The systematic review process followed a rigorous screening protocol to ensure the inclusion of studies pertinent to high-impact, low-probability (HILP) events. This process was structured to be replicable and defined around set inclusion and exclusion criteria, which were strictly adhered to in the selection of relevant literature.

#### Inclusion criteria

All selected studies had to meet each of the following criteria:

1. Focus on HILP events
  Studies were included if HILP events or HILP-like events were either the main topic or the contextual backdrop of the research. Since terminology may vary, search strings included terms conceptually equivalent to “high impact” and “low probability.” Papers that addressed these events, even if using alternative descriptors, were included. For instance, studies on ‘Black Swan’ events were considered relevant, while unrelated studies (e.g., black swan mating behaviour) were excluded.
  HILP-like events as study context
    Research focused on HILP-related topics, such as planning responses to HILP events, forecasting HILP impacts, resilience analysis for critical infrastructures or communities, and resilience forecasting, was considered relevant.
2. Relevant domain
  The study needed to fall within domains closely related to HILP events, including disaster risk reduction, risk perception and management, emergency and disaster management, organisational and operational resilience, societal resilience, system resilience, and resilience of critical services. The review encompassed financial and economic fields related to operational continuity.
3. Risk/resilience modelling or analytical methods
  If the study proposed modelling or analytical methods, these methods needed to address risk, resilience, or decision-making under uncertainty as a primary theme. For example, a study on the resilience of distribution networks under earthquake conditions would be included, while a study focused purely on landslide particle analysis without a resilience component would be excluded.

#### Exclusion criteria

The exclusion criteria were applied to filter out studies that did not align with the scope of HILP-related research:

- *Unavailable full text*—Studies were excluded if their full text could not be located despite thorough library searches.
- *Incorrect use of terminology*—Studies using search terms in unrelated contexts, such as “Black Swan” to describe animals, were excluded.
- *Irrelevant domain*—Studies focused on HILP events within non-relevant domains, such as geology, media studies, or medicine, were excluded.
- *Irrelevant outcomes*—Studies that did not address risk or resilience outcomes at the system, societal, or individual level were excluded.

#### Search terms

Search terms included a combination of terms equivalent to “High Impact Low Probability” (HILP), as well as terms equating to “high impact” and “low probability.” The search process captured literature mentioning one of the following HILP equivalents: “Black Swan,” “Grey Rhino,” “Dragon King,” “Perfect Storm,” or “Grey Swan.” Alternatively, studies with at least one “high impact” term and one “low probability” term from the following lists were also included:

- *High impact equivalents*: catastrophic, extreme risk, compound risk, concurrent risk, high loss, high consequence, high damage.
- *Low probability equivalents*: low recurrence, 100-year event, 500-year event, 1000-year event, low likelihood, rare, prototypical.

Due care was taken to interpret search results, as some terms, like “perfect storm” and “Grey Rhino,” may yield both relevant and irrelevant literature.

An initial pool of 3503 papers was identified using these search terms, with irrelevant fields removed through filters (e.g., excluding medical, biochemical, and chemical studies). Rayyan software facilitated the systematic review, with two independent reviewers assessing each paper. Any disagreements were resolved by a third reviewer with expertise in the field of risk and disaster reduction. The final screening resulted in a selection of 588 papers meeting all inclusion criteria.

### Snowballing

Based on the results of the screening, it became clear that the existing body of work on this topic is very nebulous, with large differences in terminology and a lack of common theoretical grounding. It was therefore decided that the review would better progress using a snowballing method to build upon the existing paper selection.

As sections of the review were written, papers for each section topic were selected from the 588 included papers using keyword and AI analysis. These were provided to the subject experts writing each section, who were encouraged to incorporate those they felt were appropriate for their section. The writers were also encouraged to draw upon their expertise and use snowballing where necessary to identify additional papers to include.

### Final paper review process

**Fig. 1:**
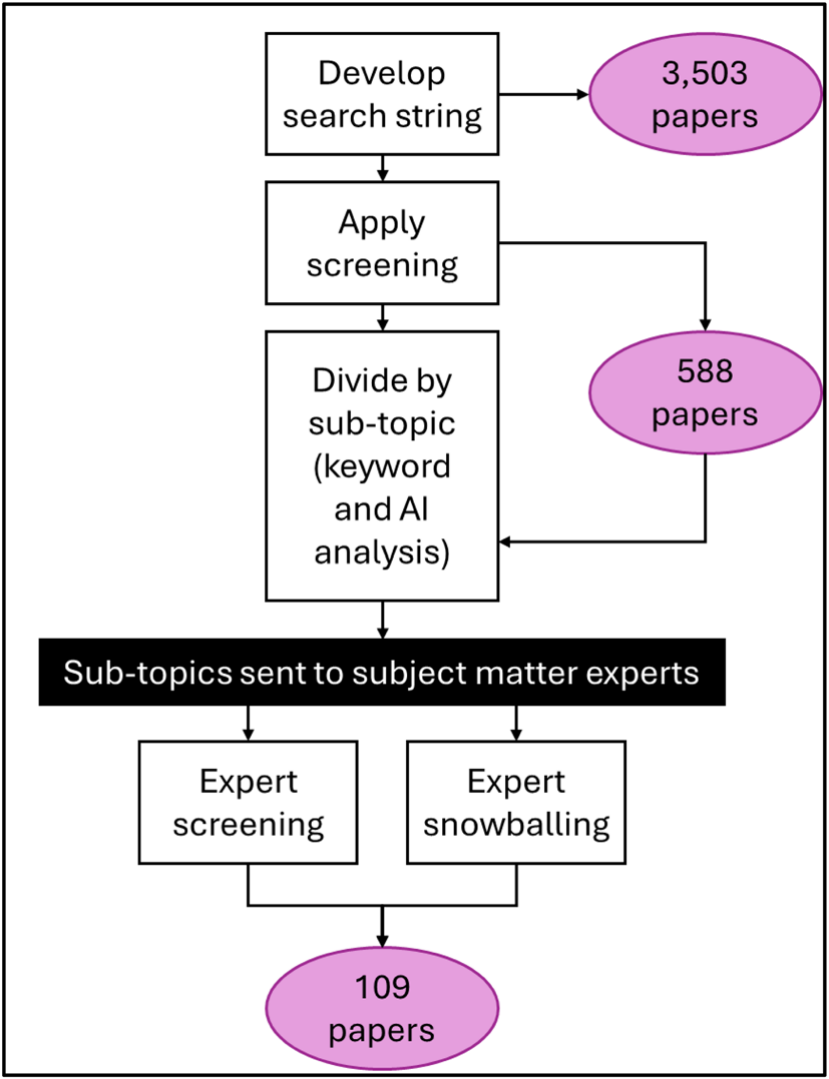
Literature selection process

Figure 1 illustrates the final paper selection process. The final result is a state of the art grounded on a systematic review but enhanced by the expertise of subject-matter experts.

### Defining HILPs and boundary conditions of the review

HILPs are an emerging topic that is still vaguely defined in the academic state of art, resulting in fragmented across subject areas and disciplines. As boundary conditions, we started from the ongoing work of the AGILE project that recently provided a definition of the subject validated by nearly 40 senior level experts affiliated to the public and private sectors (Pescaroli et al. 2025). HILPs can be considered as follows:

‘High Impact Low Probability (HILP) events are context-dependent shocks that must be assessed in proportion to the specific realities they affect. These events occur infrequently and are marked by high levels of uncertainty in both their predictability and impacts, often bringing an element of surprise or anomaly within their context. Their impact is best understood in terms of disruptions to critical services or essential social functions, where existing vulnerabilities and capacities play a crucial role in shaping the outcome and progression of the crises.

HILP events typically lack recent historical precedents, may be linked to older or geographically distant precursors, or reflect a new combination of known risks and vulnerabilities forming a “perfect storm.” When these events occur, they impact an operational environment that may have changed significantly since the last similar event, affecting both exposure and vulnerability levels.

In practice, HILPs may not meet the thresholds for mitigation within the public and private sectors due to limited resources or risk appetite. Even if the possibility of such events is acknowledged, resources might be allocated to less extreme scenarios deemed more likely.

The distinctive nature of HILPs necessitates moving beyond past lessons to better anticipate and mitigate future events, enhancing resilience to common risk patterns and vulnerabilities across different threats. Addressing these uncertainties may require adopting methodologies like lateral thinking, counterfactual analysis, adaptation pathways, the precautionary principle, and scenario stress testing.’

However, through discussions with experts, Pescaroli et al. (2025) determined that the defining characteristics of HILPs go beyond what can be captured in a few paragraphs, providing a complementary taxonomy that integrates attributes reflecting complexity, uncertainty, and recombination of known and unknown factors.

Key elements are:

- *Low probability*: The events involve compound, interacting, or interconnected dynamics (e.g., linked risks with separate but causally connected impacts).
- *Complexity*: These events may include cascading failures in highly interdependent systems (e.g., nuclear accidents) and are marked by sudden or creeping crises that are challenging to predict.
- *Recombination of known patterns*: HILPs may emerge from common hazards combined in an unusual manner within a specific context, or from interactions between physical and social dynamics
- *Context dependency*: HILP events are highly dependent on their specific contexts and must be evaluated relative to the affected environment or system.
- *Low recurrence and predictability*: These events occur with low frequency, making historical forecasting challenging due to limited precedents or distant historical occurrences.
- *Surprise or anomaly*: HILPs typically present elements of surprise or anomaly, as they do not follow regular patterns and may occur unexpectedly within certain operational contexts.
- *Critical impact on services*: The primary impacts of HILP events are measured by the disruptions they cause to critical services or social functions, which are often exacerbated by existing vulnerabilities within the affected system.
- *Combination of known and unknown risks*: HILP events often arise from a unique combination of known hazards and vulnerabilities, sometimes forming “perfect storm” scenarios that exceed traditional risk assessment approaches.
- *Lack of adequate mitigation*: Due to resource constraints or limited risk tolerance, HILPs may not meet thresholds for preventive action, requiring unconventional approaches for effective management.
- *Necessity for unconventional methods*: Given their unpredictable nature, addressing HILPs often necessitates lateral thinking, counterfactual analysis, adaptation pathways, and scenario stress testing.

## HILPs in disaster management

### Theory

Assessing risk in HILP events presents a fundamental challenge: traditional probability-based approaches struggle to accurately estimate their likelihood and impact. High-probability events tend to have well-documented recurrence rates or higher degrees of certainty in their occurrence, allowing for reasonably precise estimations of their likelihood. In contrast, the rarity of HILP events means that historical frequency is often an unreliable indicator, and the actual probability can differ significantly from observed occurrences (Velasco et al. 2023). This uncertainty is compounded by the absence of clear precursors, making these events appear sudden and unpredictable.

The low probability of HILP events can often result in them presenting as unexpected or surprise events, with government and impacted communities frequently unprepared. Unlike HIHP risks, where frequent occurrences justify investment in mitigation, HILP events are often deprioritised due to their perceived rarity. This lack of preparedness can, in turn, exacerbate their impact, as the absence of mitigation strategies can lead to greater damage and disruption when they do occur (Velasco et al. 2023). The differences in preparation for or mitigation against HILP events could offer some insight into how rare events become HILPs in some cases, whilst comparable hazards in other cases remain low impact (these low impact, low probability events can be called LILPs).

When faced with limited resources for mitigation or other preparedness measures, how these are allocated often comes down to a level of risk associated with a combination of impact and probability. This overly simple assessment of risk can misclassify HILPs as of the same risk as low impact high probability events, when the former has the potential for much more severe consequences and a high degree of uncertainty that presents a challenge for quantitative risk-based approaches (Elms 2019).

High impact events (both HIHPs and HILPs) are rarely fully costed in advance, and political and reputational costs are often neglected, which can significantly understate the true financial implications. Given the substantial uncertainty in estimating the potential costs of high impact and in estimating the low probability associated with HILP events, any estimates are more meaningful in a comparative context rather than in absolute terms (Elms 2019). Such events, even if theoretically foreseeable, have an incalculable probability and an unpredictable magnitude (Taleb 2007). The rarer an event, the more likely it is that impact assessments will be wildly inaccurate (Kyrillou 2020), leading to ineffective risk mitigation strategies. This unpredictability fosters further reluctance to invest in preparedness measures, reinforcing a cycle of vulnerability.

Public perception further complicates the issue, as research indicates that individuals often misjudge the likelihood of HILP events, either overestimating their frequency or dismissing them entirely (Sundh 2024). This cognitive bias, coupled with institutional tendencies to prioritise short-term risks, leads to systemic underestimation of rare but catastrophic events.

### Organisational preparedness

To develop strategies for managing HILPs, it is important to understand the interaction between technical vulnerabilities and their operational environments. This includes the organisational (processes, responsibilities, and institutional capacity), social and behavioural (trust, communication, cultural norms), and political and governance (legal mandates, regulatory coherence) spheres (Trump et al. 2025).

Operational environments can be complex, with multiple stakeholders, limited information, and susceptibility to misinformation. In effective crisis coordination, it is necessary to understand the interdependencies within the organisation and the connections between technical and governance aspects. Preparedness must evolve beyond asset protection to encompass the softer but equally crucial dimensions of socio-technical systems (Trump et al. 2025). These include institutional procedures, communication channels, and coordination mechanisms that, if poorly aligned or underdeveloped, can become hidden vulnerabilities or single points of failure.

Crisis conditions often reveal the fragility of systems operating under stress, whether through the burnout of frontline workers, the overextension of physical assets, or the breakdown of communication across organisational silos (Galaitsi et al. 2023; Pescaroli et al. 2021). Such failures are rarely technical alone; they are the product of deeper systemic misalignments between institutional capacity, stakeholder expectations, and operational realities.

In many cases, vulnerabilities lie not in the lack of technology but in the insufficient integration of planning and governance (Boin and Rhinard 2008). Critical infrastructures, increasingly privatised or hybrid in nature, require coordinated approaches that reconcile different organisational goals, cultures, and decision-making rhythms (Boin and Smith 2006). Yet, inter-agency collaboration frequently suffers from bureaucratic politics, misaligned incentives, or unclear lines of accountability, especially during low-attention phases when HILPs are seen as too remote to justify proactive investment. The conversion of contingency plans into operational readiness, through drills, training, and shared situational awareness, is often hindered by resource limitations, political shifts, or organisational complacency (Pescaroli et al. 2021). There is also a need to integrate societal dimensions of resilience in order to ensure an effective response ‘on the ground’ (Boin and McConnell 2007).

Slowly, emphasis is shifting to consider the complex, cross-sectoral, and trans-boundary nature of effective infrastructure system management, particularly in high-impact scenarios (Good Governance for Critical Infrastructure Resilience, 2019). However, there remains a lack of consideration of emergent and systemic risks, with many hazard-specific approaches failing to capture the emergent dynamics of HILP events.

## Risk and resilience

### Risk, resilience, and HILPS

Risk reduction targets known vulnerabilities, resilience emphasises flexibility and recovery, making it crucial for addressing HILP events. However, as these rare but potentially catastrophic events often lack clear precursors, traditional risk management alone is insufficient. By integrating resilience, disaster preparedness evolves into a proactive, hazard-agnostic approach, enabling systems to better withstand and respond to unforeseen HILPs, from global health crises to infrastructure failures.

Risk is typically understood to represent a combination of the likelihood of an event occurring and its potential negative consequences or expected losses (Kelman 2018; UNISDR terminology on disaster risk reduction|UNDRR, 2009). The concept of risk is inherently tied to vulnerability, which refers to the susceptibility of a community or system to the harmful effects of a hazard (UNISDR terminology on disaster risk reduction|UNDRR, 2009).

More recently, the significance of systemic risk in disaster management has been better understood. Systemic risks can have cascading impacts that propagate ‘within and across systems and sectors’. Such risks spread through ‘the movement of people, goods, capital and information’ within and across regional, national, and international boundaries (Sillmann et al. 2022). Increasing globalisation plays a role in the development of systemic risks, the consequences of which can include system collapse. The characteristics of system risks share many commonalities with those of HILP events, in particular the potential for widespread cascading impacts (Pescaroli and Alexander 2016).

Resilience is a dynamic concept, reflecting the capacity of a system, community, or society to withstand, adapt, and recover from hazards in a timely and efficient manner—to ‘bounce back’ from a shock or disruption (IPCC 2012; UNISDR terminology on disaster risk reduction|UNDRR, 2009). This ability to adapt and recover evolves as systems respond to changing conditions, making resilience an emerging property of complex systems (Dolan 2021; Principles for resilient infrastructure, 2022; Prothi et al. 2023). Resilience encompasses a range of factors, including social, economic, environmental, and infrastructural dimensions.

Exploration of resilience underscores the need to consider highly uncertain and consequential risk events that traditional risk management approaches can struggle to address (Linkov et al. 2016). In the context of disaster risk reduction, resilience is particularly valuable as it supports a hazard-agnostic approach to disaster management, preparing systems to deal with a wide range of potential hazards rather than focusing on specific scenarios (Beevers et al. 2022). This flexibility is crucial given the increasing unpredictability and complexity of modern hazards, which often involve multiple, interconnected risks (Warner et al. 2019).

The relationship between risk and resilience is both distinct and interconnected. Synergies between risk and resilience are evident in the development of robust systems that can anticipate, absorb, and recover from shocks (Linkov et al. 2022). For instance, reducing risk through infrastructure improvements directly contributes to greater resilience by reducing the impact of hazards. Conversely, resilience-building activities, such as community preparedness and adaptive governance, can lower overall risk by making it easier to manage and mitigate hazards when they arise.

HILP events can be considered a category of risks characterised by rarity and potentially catastrophic consequences. While traditional risk assessment can consider the nature of these type of risks, resilience is also particularly crucial in effectively managing HILPs. One key reason is that HILPs often involve unknown hazards—emerging threats that have not yet materialised or been identified through horizon scanning. Focusing solely on known risks can overlook these potentially very significant threats. By adopting a resilience perspective, even if the specific cause of disruption is unknown, the system’s capacity to recover from a HILP-level event can still be effectively evaluated and strengthened.

### Can risk registers capture HILPs?

Risk registers serve as strategic tools for governments and organisations to systematically identify, assess, and prioritise potential risks over specified timeframes. These registers provide a structured approach to understanding the likelihood and impact of various threats, thereby guiding resource allocation and mitigation strategies (Swedish National Risk Assessment, 2012 2012; National Risk Register 2023, 2023). Public versions of national risk registers, such as those from the UK, Netherlands, and Sweden, offer insights into a country’s risk landscape, though they may lack detailed methodological transparency. The stated goals of publicly available national risk registers include “to identify a range of risks that are representative of the risk landscape and can serve as a cause-agnostic basis for planning for the common consequences of risks” (National Risk Register 2023, 2023), “to give an overview of the main risks attributed to different disasters, crises and threats with potentially disrupting effects on society” (Dutch National Risk Assessment, 2019), and “to create a common understanding of serious risks … and future consensus on proposed measures and resource priorities” (Swedish National Risk Assessment 2012, 2012). Common to these objectives is the emphasis on raising awareness, fostering planning, and prioritising resources to mitigate risks effectively.

Assessment periods for public risk registers, often five years, are focused on near-term priorities. However, recent literature suggests a split approach for short- and long-term risks, typically 1–5 years and 25–35 years, respectively (Poljansek et al. 2021). This distinction allows for immediate attention to high-probability, low-impact risks, while also accommodating long-term, HILP risks driven by factors like climate change or geopolitical shifts.

Several risk registers, including the UK’s National Risk Register, (National Risk Register—2025 edition, 2025; National Risk Register 2023, 2023), employ a two-dimensional likelihood-impact matrix for assessing risks, often without precise classification of each band. The lack of agreed-upon thresholds for designating an event as a HILP risk complicates categorisation. This issue, termed “Schrödinger’s Ostrich” by Bowman et al. (2024), describes a tendency in risk management to ignore significant but theoretically possible risks. Especially for emerging or novel risks, historical data may be absent, challenging risk categorisation (Bowman et al. 2024).

While risk registers offer an important tool for awareness and risk communication, they have their limitations when it comes to HILP events. The high degree of uncertainty associated with HILPs, as well as their emergent nature and the high likelihood of compounding dynamics (Pescaroli et al. 2025), can present a challenge for hazard-specific methods like risk registers.

The assessment of systemic risks is increasingly essential as interconnected systems create cascading vulnerabilities. These complex systems require continuous evaluation to assess the potential propagation of disruptions and to identify auxiliary systems that might serve as fail-safes during crises. Contextual factors such as weather, season, and time of day also significantly influence cascading risks, underscoring the need for adaptive approaches in systemic risk assessments (Poljansek et al. 2021).

The importance of considering cascading effects is underscored by case studies such as the interdependence between power supply and IT services highlighted in the Dutch National Risk Assessment (2019). Disruptions within one sector can quickly affect others, especially as interconnected systems become increasingly reliant on digital networks and data traffic.

HILP events could be considered to represent the type of risks most likely to be overlooked or minimised in traditional risk analysis. Any risk analysis that considers HILP events, even when the method is sophisticated and detailed, should therefore be viewed and interpreted with caution (Elms 2019). To move forward with risk assessment, there is a need to move towards cross-sectoral thinking, recognising the complex dynamics between interconnected systems. Advances in remote sensing also present opportunities for dynamic decision support that is based on real-time data during extreme events (Ellingwood 2009).

### Resilience assessment

While risk assessment remains a valuable tool, there remain limitations. Though known risks can be identified, and mitigation measures implemented when necessary, residual risk will always remain (Linkov et al. 2018). Beyond this, there is an issue of completeness in all risk approaches. Risk assessments must make assumptions about what merits inclusion, and may also miss unexpected and unknown risks entirely (Elms 2019). More so than other risks, HILP events are more often unexpected and thus can be overlooked more often than known risks.

Resilience assessment, which focuses on the behaviour of the system facing disruption, rather than the hazard causing the disruption, offers a method that could overcome the incompleteness present in solely risk-based approaches. If effectively implemented, resilience assessment could address known, but unmitigated, risk, as well as improve system response to unknown or emerging threats (Linkov et al. 2018).

Traditional risk stress testing methodology encounters significant scalability issues as infrastructure systems grow in size and interconnectedness. This limitation is particularly pronounced in critical infrastructure, where the interconnected nature of complex systems can leads to compounded threats and cascading failures. The increasing interdependence of these infrastructures necessitates innovative methodologies that can effectively model how systems respond to threats and recover from disruptions. (Linkov et al. 2022). There is also increasing awareness that not all risks can be identified and appropriately mitigated against, prompting greater consideration of how systems recover from disruption in order to deliver critical services (Vugrin et al. 2011).

Resilience assessment has emerged as an approach for managing recognised but unaddressed risks, while also strengthening the system’s capacity to respond to unknown or emerging threats (Linkov et al. 2018). By enhancing adaptive capacities such as modularity, distributedness, diversity, and plasticity, critical infrastructure systems can develop greater resilience to these novel or unexpected threats (Trump et al. 2025). A recent shift towards hazard or threat-agnostic thinking opens up new doors in resilience assessment, moving the focus away from understanding the causes of disruption and towards a better understanding of system response, critical points of failure, and systemic and cascading impacts (Beevers et al. 2022; Trump et al. 2025).

Linkov et al. (2018) propose a tiered framework for resilience assessment, which categorises resilience assessment tools into three tiers. This structure allows analysts to scale assessments and associated management actions according to the scope and urgency of risks, as well as the available resources of managers seeking to improve system resilience. The framework, which is aligned with tiered risk assessment, aims to enhance the integration of resilience assessment into existing regulatory processes, streamlining its adoption and facilitating more effective risk management practices (Linkov et al. 2018).

It is important to set a solid foundation for resilience assessment by choosing appropriate metrics and standards, including those that assess a network’s vulnerability (Cutter et al. 2010), as these can guide operational planning (Kasimalla et al. 2024; Zhang et al. 2017). Metrics also allow baseline conditions to be established, facilitating the monitoring of resilience over time in a given location or system and allowing comparison across different places and systems (Cutter et al. 2010).

The value of resilience assessments for preparing for and managing HILP events has begun to be recognised for power systems, which have historically been designed to operate reliably under low-impact, high-probability disruptions (Panteli et al. 2017). Recent shifts towards resilience recognise the threat of amplifying effects from a combination of growing interdependence and environmental risks resulting from HILPs (Zhang et al. 2017). As a significant majority of disruptions to power supply systems are, directly or indirectly, as a result of weather events, a variety of methods have been considered for evaluation of power system resilience to extreme weather events (Kasimalla et al. 2024; Panteli et al. 2017; Sun et al. 2019). Research extends beyond meteorological events, with seismic events also considered (Villamarín–Jácome et al. 2019). While hazard-specific case studies are valuable for demonstrating these methods, the ultimate aim is to enhance preparedness for a broader range of HILP events.

Zhang et al. (2017) explore the intersection of power systems and natural gas systems, introducing a method to assess power system performance considering the influence of fuel supply systems. This preliminary study also identifies the need to consider cascading effects in future research (Zhang et al. 2017).

While it is promising to see steps towards resilience assessment in critical infrastructure systems, existing approaches remain largely hazard-specific and could benefit from adopting a more cross-sectoral and interdisciplinary approach (Good Governance for Critical Infrastructure Resilience, 2019). Given the complex dynamics associated with HILP events, as well as the raise of compounding and hybrid threats, a hazard-agnostic perspective offers increased flexibility and scalability when considering HILP preparedness (Trump et al. 2025).

## The compound and cascading dynamics of HILPs

HILP events rarely unfold in isolation; they propagate through interconnected systems, generating cascading and compounding failures that amplify their impact. The expansion of global interdependencies has rapidly accelerated, reshaping risk landscapes and transforming localised disruptions into systemic crises. Multiple disciplines, from sociology and network science to economics and disaster management, have examined how these complex linkages contribute to both resilience and fragility (Barabasi and Frangos 2002; Benkler 2006; Castells 1996; Dreher et al. 2008; Granovetter 1973). This section examines how these dynamics manifest across sectors, drawing on recent disasters to illustrate the vulnerability pathways through which localised disruptions escalate into systemic crises.

### Interconnected risks and the potential for cascading and compounding failures

HILPs are shaped by a web of systemic vulnerabilities that allow disruptions to cascade through multiple sectors. Historical patterns of societal collapse, particularly among advanced civilizations, have often stemmed from an inability to manage systemic interdependencies (Linkov et al. 2024). It is in modern systems, from technological networks to global supply chains, however, that the role of interconnections takes a predominant place in risk management due to the likelihood of cascading failures (Aven and Zio 2021; Timashev and Bushinskaya 2023): interdependencies can transform localised failures into widespread disruptions (Buldyrev et al. 2010). These ripple effects are characteristic of HILP events and are exacerbated by the fragility inherent in globally interconnected systems (Helbing 2013). In this regard, interdependencies between modern systems contribute to the fragility of these systems (Masys et al. 2014). This recognition has prompted warnings from organisations such as the World Economic Forum, which emphasises that ‘The world is insufficiently prepared for an increasingly complex risk environment’ (Global Risks 2015, 10th Edition, 2015) and ‘Global risks cannot be seen in isolation’ (Global Risks 2015, 10th Edition, 2015; Masys 2018). With many recent disasters resulting from unrecognized or underappreciated weaknesses in risk assessment and mitigation policies (Stein and Stein 2014), the field of disaster risk reduction has begun to focus on improving the capability of societies to cope with cascading crises and mitigate their detrimental consequences through an evolved understanding of their nature.

Cascading dynamics are driven by processes of accumulation and release concentrated in nodes within socio-technological systems (Pescaroli and Alexander 2016). When triggered, cascades recombine causes and effects, developing non-linearly through vulnerability pathways. This behaviour renders traditional models, often focused on single-point failures or individual hazards, inadequate for capturing the dynamic, multi-layered interactions that define HILP events. There is a need for a fundamental redesign of disaster risk management, able to take into account interdependencies (Helbing 2013; Masys 2018). Effective management requires moving beyond immediate triggers to examine underlying vulnerability pathways (Pescaroli et al. 2018) and the resilience potential embedded within interconnected systems (Cedergren and Hassel 2021; Liu et al. 2020; Masys 2021; Timashev 2020; Watson et al. 2022).

### Sectoral vulnerabilities to compound and cascading failures in HILP events

HILP events emerge across a vast array of sectors, where interdependencies magnify their complexity and impact. The impacts of the COVID-19 pandemic serve as a prime example, with a global health crisis cascading into economic, social, and geopolitical disruptions. Cascading and compounding dynamics disproportionately affected the most vulnerable populations, with border closures, supply chain failures, and overwhelmed healthcare systems exposing deep-seated vulnerabilities (Masys 2021; Peters et al. 2023). The compounding nature of such crises, where concurrent hazards create intersecting response challenges, exacerbates the strain on emergency management systems (Donkor et al. 2022). This was seen in the Michigan floods of 2020, where evacuation efforts clashed with public health mandates (Sohn and Kotval-Karamchandani 2023).

Environmental disasters offer further evidence of cascading failures that span natural and human systems. From Hurricane Katrina’s levee failures in 2005 (Brunkard et al. 2008; Kates et al. 2006) to the Texas freeze in 2021 (Busby et al. 2021), extreme weather events have repeatedly triggered secondary crises, amplifying their destruction. Tectonic hazards have seen similar cascades: The 2010 eruption of Iceland’s Eyjafjallajökull volcano eruption resulted in an ash cloud that disrupted European air travel (Gudmundsson et al. 2012; Kelman et al. 2023), while the 2011 Tōhoku Earthquake and Tsunami (Japan) lead to the Fukushima Daiichi nuclear disaster (Goto et al. 2021). However, not all disasters originate from a single, isolated event, many emerge from the interaction of multiple stressors (Ferrarin et al. 2021). The compound risks posed by the convergence of intense rainfall and storm surges in Hong Kong demonstrate how overlapping hazards can exacerbate disaster outcomes (Qiao et al. 2023). This underscores the urgent need for a more integrated approach to risk assessment, that accounts for the complex interplay of multiple hazards and their cascading consequences across interconnected systems.

Infrastructure networks, particularly critical energy and communication systems, are highly susceptible to cascading disruptions. Modern power grids, for instance, face growing threats from climate-induced extreme weather events, cyberattacks, and technical failures. Large-scale blackouts increasingly disrupt economies and social stability, demonstrating the urgent need for resilient power system planning (Cadini et al. 2017; Iswaran et al. 2022; Kaloti and Chowdhury 2023). Strategies to mitigate cascading failures range from complexity-based modelling (Watts and Ayala 2014) to resilience-enhancing cyber-physical power systems (Liu et al. 2020) and machine learning-based cascading failure detection (Nakas et al. 2023).

Interdependencies extend beyond power grids to water, gas, and food supply systems, where disruptions in one domain can rapidly compromise others (Paul et al. 2024). For example, a September 2013 flood in Boulder, Colorado, USA, had cascading impacts on critical food-energy-water systems (Romero–Lankao and Norton 2018). The “water-energy nexus” exemplifies fragility in interconnected systems, as failures in electric power systems often trigger cascading water distribution issues. Researchers have proposed resilience metrics (Zuloaga and Vittal 2019) and disaster severity-approach (Alhazmi et al. 2022) to network evaluation to address these vulnerabilities. Similarly, power system and natural gas system are strongly interconnected with the potential risk of failure propagation and the amplification of disruptive effects. Research has considered the interdependence of natural gas and electricity networks when affected by multi-hazards such as hurricane and earthquakes (Ravadanegh et al. 2021), as well as the challenge of modelling power system resilience considering associated fuel supply (Zhang et al. 2017).

The industrial sector also faces increasing risks from cascading disasters. Chemical accidents, energy infrastructure failures, and industrial process disruptions have all contributed to large-scale crises, necessitating more integrated risk management approaches (Cozzani and Reniers 2021). Furthermore, the rise of digital technologies has introduced cyber vulnerabilities into critical infrastructure systems, raising concerns about the potential for simultaneous failures across networked systems (Smith 2014).

Beyond infrastructure, global food security is threatened by cascading and compounding crises. Climate change, economic shocks, and political instability have led to widespread disruptions in food production and distribution. Environmental stressors, combined with the impacts of climate change, can have cascading impacts on agriculture and fisheries and require mitigation and strategic adaptation to reduce societal vulnerability (Thiault et al. 2019). A recognition of vulnerability pathways is necessary to understand how to reduce risks in global food systems (Fan et al. 2021).

Finally, financial markets exemplify the interconnected dynamics of HILP events. The 2008 financial crisis, precipitated by the collapse of the housing market, rapidly cascaded through global economies due to the deeply interwoven nature of financial institutions (Haldane and May 2011). A better understanding of how cascading failures can propagate through complex networks of this nature can allow for better planning and potential avoidance of particularly devastating impacts (Faggini et al. 2019).

## Individuals and communities

Laypeople’s risk perceptions are often deemed irrational compared to expert assessments, but this is not entirely accurate. While experts focus on technical estimates of fatalities, laypeople consider broader factors like potential catastrophes, controllability, and threats to future generations (Aven and Zio 2021). This difference highlights a qualitative disparity in risk perception, suggesting that risk is a subjective construct shaped by human cognition and culture, influencing all stages of risk assessment and communication.

In this sense, to better understand the way people perceive risk and why certain risks are deemed more acceptable than others, Slovic et al. (1984) developed the psychometric paradigm to systematically categorize risks based on individual or group perceptions. This framework identifies two key factors: “dread risk”, associated with fear, catastrophic potential, and inequitable risk–benefit distribution, and “unknown risk”, involving unobservable, novel hazards with delayed harm. Laypeople’s risk assessments align closely with these factors. HILP events, characterised by high dread and unknown risk, are perceived as highly threatening due to the emotional impact of potential catastrophic consequences, while the low probability of occurrence makes accurate risk assessment difficult.

### Low-probability and human cognition

High-impact events often prompt individuals and communities to support risk regulations and comply with prevention measures. However, low-probability events are frequently neglected as they are not known or considered in the first place (Sundh 2024). Conversely, following a catastrophe, emotionally charged, vivid outcomes and media images often lead to an overestimation of the true risks of similar events (Cox 2023). This can result in under-preparation before an event and overreaction afterwards, highlighting the need to better understand the psychological mechanisms behind risk information processing and human behaviour.

#### Risk underestimation and probability neglect

Literature extensively documents cognitive biases and heuristics that lead to risk-taking behaviour in face of critical HILPs. Individuals often seek information that confirms their existing beliefs, avoid unpleasant information, and prefer certainty, resulting in a lack of consideration for these risks (Rheinberger and Treich 2017). For instance, homeowners in flood-prone areas frequently avoid purchasing insurance (Botzen and van den Bergh, 2012; Kunreuther and Pauly 2004), and building owners downplay earthquake mitigation measures compared to engineers (Meszaros 2005). Similarly, farmers exhibit risk-loving behaviour towards monetary losses, resulting in a low willingness to pay for weather shock protection (Duden et al. 2023). In organizations, similar patterns are seen as “organizational myopia” leads to tunnel vision and unexpected surprises due to psychological and organizational barriers (Feduzi et al. 2022).

The availability heuristic can explain this in the sense that individuals assess the likelihood of an event based on how easily relevant instances can be recalled (Tversky and Kahneman 1973), which is influenced by aspects such as media coverage, interest groups/individuals and personal experiences (Lichtenstein et al. 1978). Since HILPs are infrequent, they are perceived as less likely, which fosters inadequate preparedness; but they can also be perceived as more likely if made ‘available’ to the mind. This is because people tend to be more concerned about risks that are ‘available’ to the mind—those that are recent, visible, and/or evoke strong imagery. This often leads to regulation that is reactive, implemented only after an event has occurred. For instance, people may worry more about airplane accidents than car accidents because the former are dramatic and newsworthy, while the latter are commonplace. This heuristic explains why public concern for rare catastrophic risks is often low. This disconnect can result in political neglect of such risks, exacerbated by factors like short-term political cycles, collective action problems, and varying cultural attitudes towards global catastrophes (Wiener 2016).

Other than the availability heuristic, there are numerous biases that lead to misallocations of catastrophe risk management concerns and resources (Cox 2023; Sundh 2024). Some of these biases include: (a) the optimistic bias, which is the conviction that one is less likely than others to experience negative events; (b) the status quo bias, which is the preference for preserving the status quo over making changes; (c) indecision, procrastination, and an excessive aversion to acting on uncertain probabilities (ambiguity aversion); (d) distorted incentives to take care, such as agency effects, moral hazard, and free riding; (e) imperfect learning and social adaptation heuristics, such as herd-following, group-think and mass numbing; (f) distributed responsibility in carrying out preparedness measures and responses; and (g) loss aversion in prevention, as individuals tend to prefer avoiding losses over acquiring equivalent gains.

Having this in mind, expert assessment is essential to address probability neglect and ensure that uncommon risks receive the attention they deserve. Much risk regulation is spurred by policy learning from experience and experimentation, but rare threats do not allow for such adaptive learning. This absence of learning opportunities underscores the need for a precautionary approach. Foresight and anticipation are crucial in preventing rare catastrophic risks, emphasizing the importance of resilience building to mitigate potential disasters effectively.

#### Risk overestimation and excessive preparation

HILPs such as nuclear disasters or terrorist attacks often evoke strong emotions, skewing public perception of their frequency and severity. This strong emotional response can overshadow statistical realities, leading to an overestimation of such risks. This is related to the affect heuristic, which involves making decisions based on emotions rather than facts. Slovic’s 1987 study highlights how emotions interfere with rational risk evaluation (Slovic 1987). Furthermore, emotional responses to risk are influenced by several factors, notably pre-existing beliefs and cognitions (Dennis et al. 2021).

The recency effect, a consequence of the availability heuristic, suggests that recent experiences with disasters make individuals more likely to feel at risk and take preventative action, such as higher insurance purchases following disasters (Botzen and van den Bergh, 2012). Media portrayal of risks is often sensationalised and can skew public perception and fear, leading to an overestimation of rare events’ frequency. This can result in disproportionate attention and resources being allocated to certain risks, such as terrorism, while less socially amplified risks, such as the case of pandemics before the outbreak of COVID-19, may be overlooked. This misallocation can impact public policy and resource distribution, perpetuating a cycle of over-regulation for some risks and negligence for others (Rheinberger and Treich 2017).

Additionally, it is important to keep in mind that severe life events leave lasting impressions that significantly impact physical, mental, and community health. These events are often related to trauma, shock, post-traumatic stress disorder, depression, anxiety, social tension, substance abuse, aggression, violence, and the loss of place identity, autonomy, and control (Clayton et al. 2021). Therefore, in addition to considering insights that help risk management teams overcome systemic inertia and political indifference, a comprehensive crisis and risk communication strategy should be incorporated that fosters preparedness and recovery under high environmental and social stress to foster individual and community resilience.

### Implications for risk and crisis communication

As reflected above, when it comes to communicating risk regarding HILP events, it is also imperative to have in consideration people’s risk perception and beliefs to communicate risk efficiently; otherwise, risk communication will not achieve the desirable outcomes.

One relevant example of these communication failures happened during the COVID-19 pandemic. Initially, it was believed that providing clear and understandable information would suffice. However, experts today know that risk communication must account for psychological, emotional, cultural, social and ethical aspects to be comprehensive (González et al. 2014). Effective risk communication during the pandemic faced significant challenges that can be attributed, namely, to the evolving nature of scientific knowledge about the virus, the complexity of the pandemic itself, and the inherent uncertainties surrounding the virus and its spread. Despite efforts to communicate, these complexities often led to unclear messaging that failed to resonate with the public. One of the central issues was the difficulty in addressing the diverse concerns and perspectives of different societal groups. Public health measures such as social distancing, mask-wearing, and restrictions on public life were essential for mitigating the spread of the virus. However, these measures often required individuals to make personal sacrifices with uncertain immediate benefits, particularly if they did not perceive themselves to be at high risk or if they were sceptical of the information presented—something that becomes more likely the lower the probability of the event. Effective risk communication relies heavily on public trust in authorities and the alignment of public behaviour with recommended guidelines. During the pandemic, trust in authorities was often strained due to conflicting messages, politicisation of health recommendations, and perceived inequalities in the distribution of burdens and benefits associated with protective measures (Palma-Oliveira et al. 2021).

Addressing these challenges requires a multidisciplinary approach to help in crafting messages that are not only scientifically accurate, but also resonate with diverse belief systems and cultural contexts. Tailoring communication strategies to different groups and articulating clear risk-reward scenarios are crucial steps in fostering understanding and compliance. Moreover, successful risk communication necessitates the active involvement of trusted stakeholders, including governmental bodies and community leaders who can effectively convey information and encourage behavioural change. By acknowledging and addressing the concerns of various groups, and by promoting transparency and inclusivity in communication efforts, the effectiveness of risk communication can be significantly enhanced (Palma-Oliveira et al. 2021).

Openness, transparency, dialogue and participation are key features of effective risk management and communication frameworks (Aven and Zio 2021). Furthermore, both before and during a crisis, uncertainty is often a major obstacle in risk communication. Effective decisions require risk assessment, but gaps in knowledge are common so, until a complete evaluation is available, it is essential to acknowledge uncertainties by saying “we don’t know”. Discussing possible unexpected crises is necessary, and risk communication should recognize and honestly convey the uncertainty of HILP events (González et al. 2014). For this, a cooperative approach is crucial, reflecting a three-way process that includes interactions among scientists, between scientists and public authorities, and between scientists and the public (González et al. 2014).

Effective risk communication strategies should also be tailored to the levels of risk and public perception. For instance, if a situation/threat is characterised as high risk, but associated with low risk perception, efforts should focus on promoting awareness of the danger so that it is well understood, and the recommended actions can be carried out. If the risk perception is somehow balanced, interpersonal dialogue, supplemented by messages through specialised media, is most effective. However, if there is low risk, but an elevated risk perception, messages should aim to reduce unnecessary alarm and explain the problem realistically. In crisis situations, communicators must articulate clear and precise messages explaining what is happening and how to act. The increasing manipulation of information is a significant concern, as it can confuse citizens or lead to indecisiveness during a crisis. In this sense, controlling the spread of false information online about natural hazards and rapidly publishing accurate information should be a priority (González et al. 2014).

In broader terms, enhancing societal resilience when it comes to HILP events requires a broad awareness of potential catastrophes, challenging the mentality that “it couldn’t happen here”. It also requires ensuring that essential response mechanisms are in place and functioning effectively, preparing first responders, raising awareness of potential risks, investing in resilience, strengthening governance frameworks, and considering socio-economic factors (Boin and McConnell 2007). It is important to keep in mind that, during crises, there is a societal expectation for government authorities to take charge and restore order. Citizens, media representatives, lobby groups, public administrators, and private organizations all look to the government for leadership and decision-making to reestablish normalcy. Consequently, effective leadership is crucial for facilitating an operational response (Boin and McConnell 2007).

While past crisis highlight numerous challenges in risk communication, they also underscore the importance of adapting strategies to the specific needs, expectations, and belief systems of different groups. By fostering tailoring of messages, transparency, trust and alignment, crises can be managed and communicated more effectively, even amidst uncertainties as profound as those caused by an HILP event.

## Conclusions

The academic state of art on the HILP events has evolved significantly in recent years. Establishing a clear and unified understanding of what constitutes a HILP event and what is meant by system resilience will underpin a more cohesive research trajectory. An improved consistency will facilitate the comparison of findings and the sharing of lessons learned.

Our review of the state of the art highlighted some existing strengths and weaknesses in the field. As the research evolves, the first element to consider is that there is still a tendency of the literature to be “siloed”. HILPs have been analysed in modelling approaches, statistical analysis, impact assessments, and retroactive cases that interlink root causes and effects, yet often within single-hazard domains such as seismic risk or flood management.

Additionally, while risk perception is emerging as a relevant field, research on the organisational implications of HILPs, and how findings can inform effective governance and preparedness, remains underdeveloped. Notably, HILPs are well codified for crisis and disaster response (after impact, once likelihood uncertainty is removed), while applications in pre-event training and strategy remain limited. This reflects the long-standing dilemma in disaster risk reduction, where political capital flows more readily toward response than preparedness. This gap requires urgent attention given the shifting risk landscape.

The growing body of resilience assessment methods, especially in power systems, provides a valuable foundation for addressing these weaknesses. This work recognises the threat of HILPs and demonstrates how resilience assessment can support both preparation and mitigation. Looking forward, there is a need to apply these resilience assessment approaches to other sectors and across interconnected systems, offering deeper insights into managing the complex risks associated with HILP events across various infrastructures. Moreover, addressing cascading risks and the interactions between different sectors is crucial for a comprehensive understanding of systemic risk. Ultimately, resilience is essential for a hazard-agnostic approach to disaster management, enabling systems to respond effectively to a broad spectrum of potential threats. Considering natural hazards, for example, this approach shifts the focus from the triggering event itself (e.g. flooding) to the common elements between two different triggers (e.g. flooding triggered by storm and landslide). By incorporating a focus on resilience, efforts in disaster risk reduction can ensure that systems are not just protected against known risks but are also adaptable to unforeseen challenges.

Looking forward, integrating interdisciplinary perspectives and scenario planning can enhance the ability of individuals, communities, organisations and nations to anticipate and mitigate the impacts of HILP events. As society’s critical systems grow increasingly interconnected, bringing together the threads of risk and resilience offers a path toward more secure and sustainable interconnected systems in the face of disasters.
